# Doxycycline inhibits experimental cerebral malaria by reducing inflammatory immune reactions and tissue-degrading mediators

**DOI:** 10.1371/journal.pone.0192717

**Published:** 2018-02-13

**Authors:** Kim E. Schmidt, Janina M. Kuepper, Beatrix Schumak, Judith Alferink, Andrea Hofmann, Shanshan W. Howland, Laurent Rénia, Andreas Limmer, Sabine Specht, Achim Hoerauf

**Affiliations:** 1 Institute of Medical Microbiology, Immunology and Parasitology, University Hospital Bonn, Bonn, Germany; 2 Department of Psychiatry and Psychotherapy, University Hospital Muenster, Muenster, Germany; 3 Singapore Immunology Network, Agency for Science, Technology and Research (A*STAR), Singapore, Singapore; 4 Clinic for Anaesthesiology and Intensive Care, University Hospital Essen, Essen, Germany; 5 Institutes of Molecular Medicine and Experimental Immunology, University Hospital Bonn, Bonn, Germany; Instituto Oswaldo Cruz, BRAZIL

## Abstract

Malaria ranks among the most important infectious diseases worldwide and affects mostly people living in tropical countries. Mechanisms involved in disease progression are still not fully understood and specific treatments that might interfere with cerebral malaria (CM) are limited. Here we show that administration of doxycycline (DOX) prevented experimental CM (ECM) in *Plasmodium berghei* ANKA (PbA)-infected C57BL/6 wildtype (WT) mice in an IL-10-independent manner. DOX-treated mice showed an intact blood-brain barrier (BBB) and attenuated brain inflammation. Importantly, if WT mice were infected with a 20-fold increased parasite load, they could be still protected from ECM if they received DOX from day 4–6 post infection, despite similar parasitemia compared to control-infected mice that did not receive DOX and developed ECM. Infiltration of T cells and cytotoxic responses were reduced in brains of DOX-treated mice. Analysis of brain tissue by RNA-array revealed reduced expression of chemokines and tumour necrosis factor (TNF) in brains of DOX-treated mice. Furthermore, DOX-administration resulted in brains of the mice in reduced expression of matrix metalloproteinase 2 (MMP2) and granzyme B, which are both factors associated with ECM pathology. Systemic interferon gamma production was reduced and activated peripheral T cells accumulated in the spleen in DOX-treated mice. Our results suggest that DOX targeted inflammatory processes in the central nervous system (CNS) and prevented ECM by impaired brain access of effector T cells in addition to its anti-parasitic effect, thereby expanding the understanding of molecular events that underlie DOX-mediated therapeutic interventions.

## Introduction

Malaria is caused by the vector-borne transmission of *Plasmodium* ssp. parasites and remains one of the major infectious diseases in the world. The disease constitutes not only a major health burden but also has negative socio-economic consequences, especially in developing sub-Saharan countries. Of the five human pathogens, *P*. *vivax*, *P*. *malariae*, *P*. *ovale*, *P*. *knowlesi* and *P*. *falciparum*, the latter is clinically the most relevant parasite. *P*. *falciparum* causes malaria tropica, which is often characterized by severe pathologies such as severe anaemia, respiratory distress, organ failure or cerebral malaria, subsequently leading to coma and death especially in children under the age of five years [1]. It is widely accepted that CM is not only caused by sequestration of parasitized red blood cells but also is a result of overwhelming inflammatory responses of the host that trigger additional effector mechanisms leading to immune-mediated pathology [2]. However, the molecular mechanisms and detailed processes during disease progression to cerebral malaria are not fully understood. Evaluation of the infection in humans is very limited i.e. to the analysis of blood-samples and of post-mortem material due to ethical reasons.

The mouse model of experimental cerebral malaria (ECM) using *Plasmodium berghei* ANKA (PbA) for infection of susceptible C57BL/6 mice is a well-established and valuable tool for analysing the molecular mechanisms leading to this disease, since several processes are similar to those occurring in human cerebral malaria [3–5].

Pro-inflammatory processes involving several mediators have been experimentally identified to play a role in *Plasmodium ssp*. infection-induced pathology during ECM and emphasise the complexity of the disease [5, 6]. Immune reactions during *Plasmodium* infection include the activation of antigen-presenting cells (APCs) such as dendritic cells (DCs) and macrophages, which recognize and take up the infected erythrocytes, parasites or parasite-derived particles [7–9]. Subsequently, pro-inflammatory cytokines are released, which also serve as a first anti-infective defence. Parasite-derived antigens are taken up and presented in order to activate effective parasite-specific immune responses [10]. In addition, tissue activation occurs due to the extensive production of pro-inflammatory cytokines like TNF and IFN-γ signature cytokines during *PbA* infection. These overwhelming inflammatory processes apparently aid parasite clearance, but also pose the risk of damaging bystander tissue and breakdown of the BBB, thereby resulting in detrimental consequences [11]. Importantly, *Plasmodium* infection and CM can be prevented and treated. The synthetic tetracycline doxycycline (DOX) is an approved antibiotic exhibiting anti-parasitic properties and ranks among approved antimalarial drugs [12]. DOX targets the parasite-specific organelle apicoplast, a vestigial plastid derived from a former endosymbiont and which is essential for survival of the parasite. With a delay of 48 hours, DOX efficiently inhibits the protein synthesis of apicoplast-encoded genes, thereby leading to an impairment of the apicoplast’s function [13]. Due to this delayed effect on parasite replication DOX is rather a valuable tool for chemoprophylaxis but not qualified as a fast-acting therapeutic anti-malaria agent [14]. Therefore, for treatment of severe malaria, the combined intravenous application of artemisinin together with tetracycline, DOX or clindamycin has been omitted some time ago [15, 16]. For the treatment of severe malaria in order to achieve rapid parasite elimination the World Health Organization (WHO) recommends the use of artemisinin-based combination therapies (ACT). The current guidelines for malaria treatment recommend the use of DOX solely for follow-up treatment in support of quinine- and artemisinin-based therapies and prophylaxis [16] due to its anti-parasitic actions.

Interestingly, besides anti-microbial and anti-parasitic functions, tetracyclines also exert immunomodulatory properties, as has been shown for DOX in diseases such as abdominal aneurysm, periodontitis, experimental autoimmune encephalomyelitis (EAE) and focal ischemia [17–21].

The aim of our study was to analyse the therapeutic effect and immune-modulating properties of DOX with regards to the development of ECM during an already on-going PbA infection. We show that DOX treatment successfully prevented ECM and enhanced the survival of PbA-infected mice. Our analysis revealed that inflammatory immune responses and T cell infiltration were markedly reduced in brains of DOX-treated PbA-infected mice that contained large numbers of activated T cells in their spleens. These new observations in regard to the additional anti-inflammatory properties of DOX might be useful for the development of new treatment strategies and could broaden therapeutic options.

## Results

### Intravenous administration of DOX prevented ECM of *P*. *berghei* ANKA infected mice

**Fig 1:**
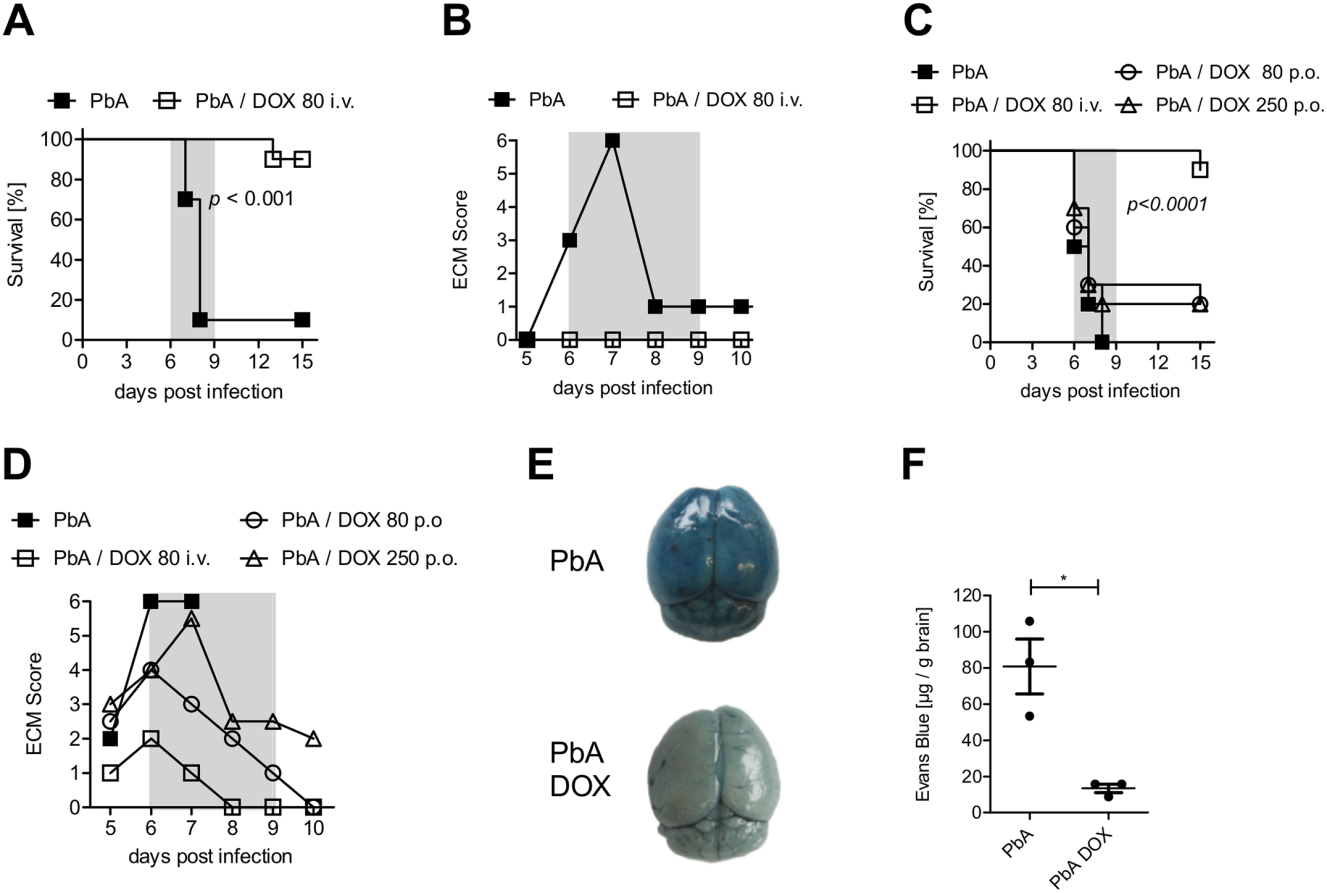
DOX administration reduced ECM development and stabilized the blood-brain barrier after PbA infection. (A, B) C57BL/6 mice received 5*10^4^ *PbA*-infected erythrocytes (PbA-iRBC) and indicated mice were injected i.v. with 80 mg/kg DOX dpi 4–6. All animals were then monitored for (A) survival and (B) ECM score (ECM phase dpi 6–9 marked in grey). (C, D) C57BL/6 mice received 5*10^4^ iRBC and indicated groups of mice were treated orally with either 80 mg/kg/day or 250 mg/kg/day from dpi 4–6. Mice treated i.v. with 80 mg/kg/day DOX (dpi 4–7) served as reference treatment group. All mice were monitored for (C) survival and (D) ECM score (ECM phase dpi 6–9 marked in grey). Data sets are representative for 2–3 individual experiments with n = 10 mice/group. Survival data were analyzed with log-rank (Mantel-Cox) test. ECM scores are displayed as median. (E, F) Mice were infected with 5*10^4^ PbA-iRBC ± 80 mg DOX/kg/day. The integrity of the BBB was analyzed with an Evans Blue assay on dpi 6. All mice were injected i.v. with 2% Evans Blue dye and one hour later, extravasation of the dye into the brain was determined. (E) Photo documentation of the discoloration and (F) quantification of dye extravasation into the brain by measuring the absorbance of brain tissue at 620nm. Data show representative results of 1 from 2 independent experiments. *p<0.05, t-test on data after analysis of normal distribution.

We addressed the question whether the antibiotic DOX might influence the outcome of PbA infection and ECM in C57BL7/6 mice. For this, we infected C57BL/6 mice with *P*. *berghei* ANKA parasitized red blood cells and confirmed successful infection of the mice (Figure A in S1 Fig). To study the effect of DOX on the course of ECM, we administered in accordance to pharmacokinetic studies from Prall et al. 80 mg/kg DOX per mouse intravenously (i.v.) on a daily basis from day 4 until day 6 *post* PbA infection (dpi 4–6) [22]. Ninety percent of untreated PbA-infected control mice developed ECM (dpi 6–9), whereas all DOX-treated PbA-infected mice were completely protected from ECM and survived this period (Fig 1A and 1B). As expected, parasitemia was reduced in blood and brain tissue of PbA-infected mice that were treated with DOX (Figures B and C in S1 Fig). Stopping the treatment after 6 dpi led to hyperparasitemia (Figure D in S1 Fig) or anaemia in DOX-treated animals around dpi 20 and therefore mice were sacrificed according to predefined ethical criteria.

In contrast to i.v. injection of DOX, oral administration of either the same dose (80 mg/kg) or an elevated dose of 250 mg/kg given daily from dpi 4–6 did not influence ECM progression, nor did it improve the survival of PbA-infected mice (Fig 1C and 1D). Subsequent experiments were performed with i.v. injection of 80mg DOX/kg from dpi 4–6. Thus, we could show that i.v. administration of DOX prevented efficiently ECM in already infected mice.

### BBB integrity was intact after DOX treatment

We next investigated whether brains of DOX-treated mice that were protected from ECM showed differences in the stability of the blood-brain barrier (BBB), since a BBB disruption, increased vascular permeability and the accumulation of activated T cells in the brain have been considered to be important prerequisites for the development of ECM. Therefore, vascular leakage into brain tissue was visualized and quantified with Evans Blue dye. Brains of ECM-positive PbA-infected mice exhibited on dpi 6 strong coloration and enhanced extravasation of Evans Blue (Fig 1E and 1F). In contrast, brains of DOX-treated mice were marginally stained and the concentration of Evans Blue dye in the brain tissue was low (Fig 1E and 1F). Thus, DOX-treated mice successfully maintained the integrity of the BBB, which corresponded with the absence of ECM-induced pathology.

### DOX altered inflammatory responses and the activity of tissue degrading enzymes in brain tissue of ECM-negative PbA-infected mice

We then studied whether immune responses in the CNS and in the periphery were altered in DOX-treated mice that were protected from ECM. Interleukin 10 (IL-10) is a key anti-inflammatory factor associated with the capacity to control pathology also in ECM [23]. To study the role of IL-10 in DOX-induced protection, we investigated IL-10 deficient mice. Importantly, PbA-infected IL-10-deficient animals were still protected from ECM after DOX treatment, which clearly excluded any role of this strong anti-inflammatory cytokine in this setting (Figure E in S1 Fig). For further investigation of protective or alternatively activated immune reactions induced by DOX resulting in prevention of ECM-related pathology, we extracted RNA from the brains of naïve mice, PbA infected WT mice and from DOX-treated PbA-infected WT mice to perform a PCR-array. Brains from control-infected B6 mice without DOX treatment revealed an enhanced gene expression of well-described effector molecules in ECM such as chemokines (CCL2, CCL5, CXCL1, CXCL2), adhesion molecules (ICAM1) and cytokines (IL-6 and TNF) in the brain tissue of dpi 6 infected mice in comparison to brains of naïve mice (S1 Table).

**Fig 2:**
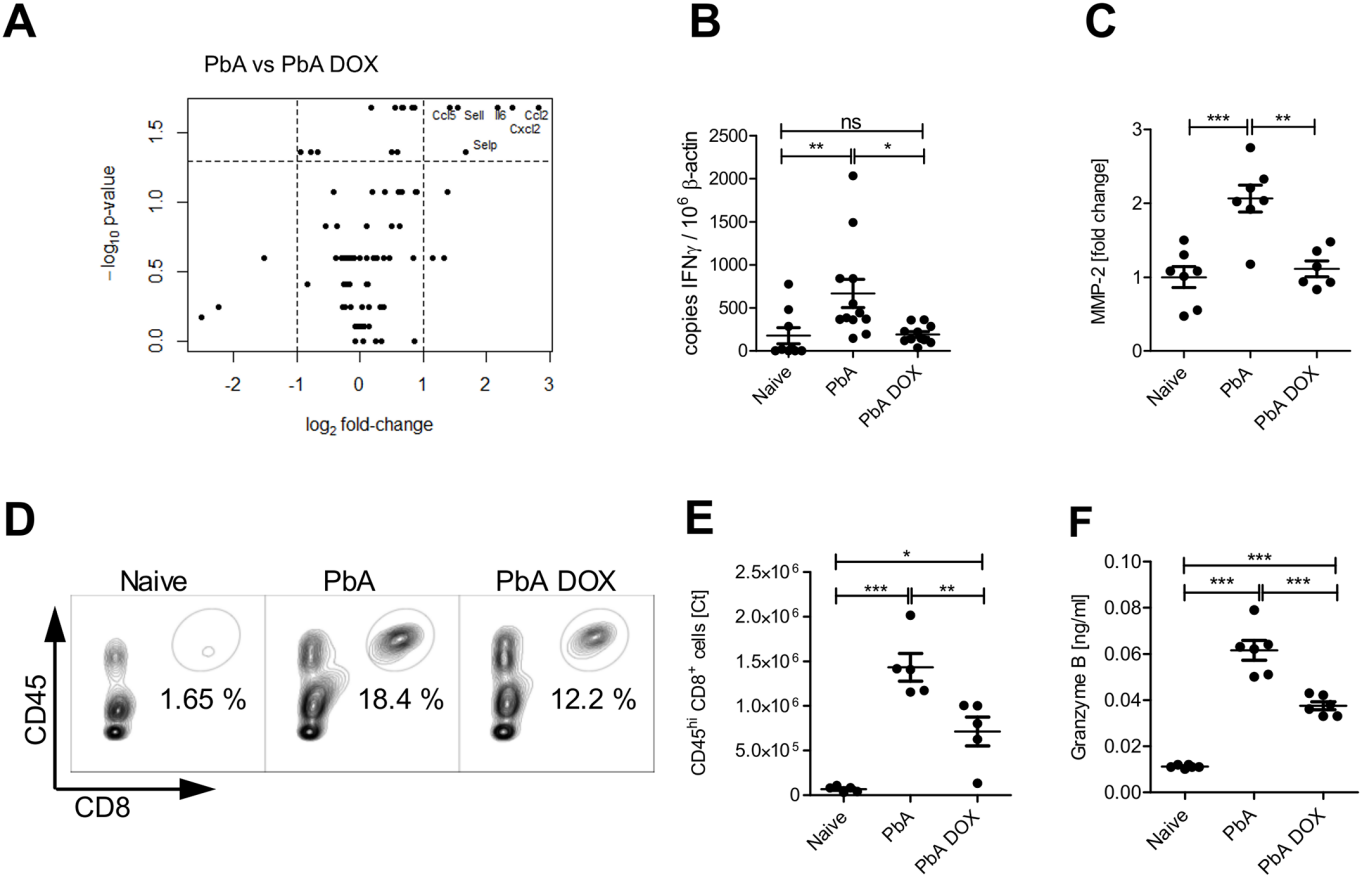
DOX regulates inflammatory responses and tissue degrading enzyme activity in brain cells of PbA infected animals. C57BL/6 mice were left naïve or infected with PbA as described above and indicated groups received DOX (80 mg/kg/day) from dpi 4–6. Six days post PbA infection, brain tissue of naïve mice and PbA infected mice ± DOX was examined for inflammatory responses and MMP activity. (A) RNA was prepared from brain tissue and analyzed via PCR array. Volcano plot analysis of the comparison PbA versus PbA DOX: Shown is the logarithmic fold-change (x-axis) against the negative logarithmic p-value (y-axis). The dashed lines indicate where p ≤ 0.05 and FC ≥2. (B) Additionally, IFN-γ was analyzed by RT-qPCR in the brain tissue of all groups (C) Quantification of MMP-2-specific zymography. Brain tissue extracted on dpi 6 was subjected to zymography and quantified by scanning densitometry of the gelatinolytic bands of MMP-2. Representative experiments are shown with 6–7 mice/ group. Relative scanning units of MMP-2 are shown as fold change against expression of naïve animals displayed as median (D) Characterization of brain leukocytes on dpi 6 from naïve, PbA and PbA+DOX-treated animals. Flow cytometry gating scheme to identify CD8^+^T cells is shown for representative animals from naïve and infected ± 80 mg/kg/day DOX mice. (E) Absolute numbers of brain infiltrating CD45^hi^CD8a^+^ cells as determined by FACS. (F) Brain tissue supernatant from dpi 6 was analyzed with an ELISA for granzyme B. (B, C, E, F) *p<0.05, **p<0.01, ***p<0.001, ANOVA with Tukey’s post test on data after analysis of normal distribution.

Importantly, a comparison of PbA-infected mice *versus* DOX-treated PbA-infected animals revealed reduced gene expression of several inflammatory mediators in the brain tissue of DOX-treated mice. The most prominent down-regulation was detected in IL-6 and in a group of chemokines, in particular CCL2, CXCL2 and CCL5 (Fig 2A).

Furthermore, the volcano plot revealed in brain tissue of DOX-treated mice a significantly reduced expression of adhesion molecules L-selectin and P-selectin (Fig 2A), which have been associated with pathogenesis of severe malaria [24]. Although we had observed reduced gene expression of CCL5, which is together with IFN-γ associated with cerebral malaria in humans and mice, protein levels of this mediator were reduced in supernatants from brain tissue cultures from DOX-treated mice and control-infected mice, but the difference was not statistically significant (Figure A in S2 Fig). In contrast, mRNA levels of IFN-γ measured by RT-qPCR in brain tissue of the respective groups was significantly elevated in PbA-infected mice but not in those treated with DOX after infection (Fig 2B). Supernatants of brains from both PbA-infected groups contained enhanced levels of TNF family proteins compared to those from naïve animals, but DOX-treatment reduced the extent of this increase significantly (Figure B in S2 Fig). TNF is relevant for the induction of metalloproteinases, which are important molecules involved in tissue degradation and destruction [25]. To analyse their activity in the brains of PbA-infected mice in comparison to brains of naïve or control-infected mice, we isolated gelatinolytic matrix-metalloproteinases 2 and 9 (MMPs) from brain tissue of all groups of mice to perform a zymography assay. We detected enzymatic degradation of gelatine in SDS gels in all three groups. While in brains of DOX-treated mice MMP-2 levels were significantly reduced in comparison to brains of untreated control-infected mice (Fig 2C), MMP-9 activity was unaffected by DOX treatment (Figures B and C in S2 Fig).

### DOX prevented cellular influx into the brains of PbA-infected animals

As we had observed a stabilized BBB and less production of inflammatory molecules in DOX-treated mice, we hypothesized that DOX-treatment interfered with the recruitment of detrimental effector cells to the brain. Therefore, we analysed the cellular CD45^hi^ infiltrates and production of effector molecules in the brains of the DOX-treated *versus* untreated mice. Granzyme B represents an important molecule involved in tissue damage and cell destruction, which is essential for the development of ECM [26]. Among the CD45^hi^ cells, DOX-treated mice contained fewer CD8^+^ T cells (Fig 2D and 2E). The increased frequency and gMFI of intracellular granzyme B from these CD8^+^ T cells that was observed after PbA-infection did not change after DOX treatment (Figures E and F in S2 Fig). However, we found significantly lower levels of secreted granzyme B in by collagenase-digestion isolated brain cells from DOX-treated PbA-infected mice compared to cells isolated from PbA control-infected mice (Fig 2F).

It has been shown that cross-presentation of parasite-specific peptides by brain microvessels to parasite-specific cytotoxic T cells is a key determinant of ECM development [27]. Therefore, we analysed whether DOX alters local cross-presentation in the brain, which subsequently could influence the cytotoxic activity of brain-infiltrated CD8^+^ T cells. After DOX treatment, the capacity of brain microvessels to cross-present PbA-derived antigen to TCR-transduced NFAT-lacZ reporter cells was slightly decreased compared to that from untreated PbA infected animals, but did not reach statistical significance (Figure G in S2 Fig).

Our data indicate that DOX dampened infection-induced inflammatory processes in the brain of PbA-infected mice, whereas PbA infection in untreated mice triggers brain inflammation, characterized by recruitment of effector cells, activity of tissue-degrading enzymes and secretion of soluble inflammatory mediators. We conclude that DOX treatment reduced such infection-induced responses on the mRNA level as well as on protein level and enzymatic activity, thereby ameliorating inflammation in the brain.

### Increased numbers of CD8^+^ T cells and decreased lytic activity in the spleens of PbA infected mice after DOX treatment

**Fig 3:**
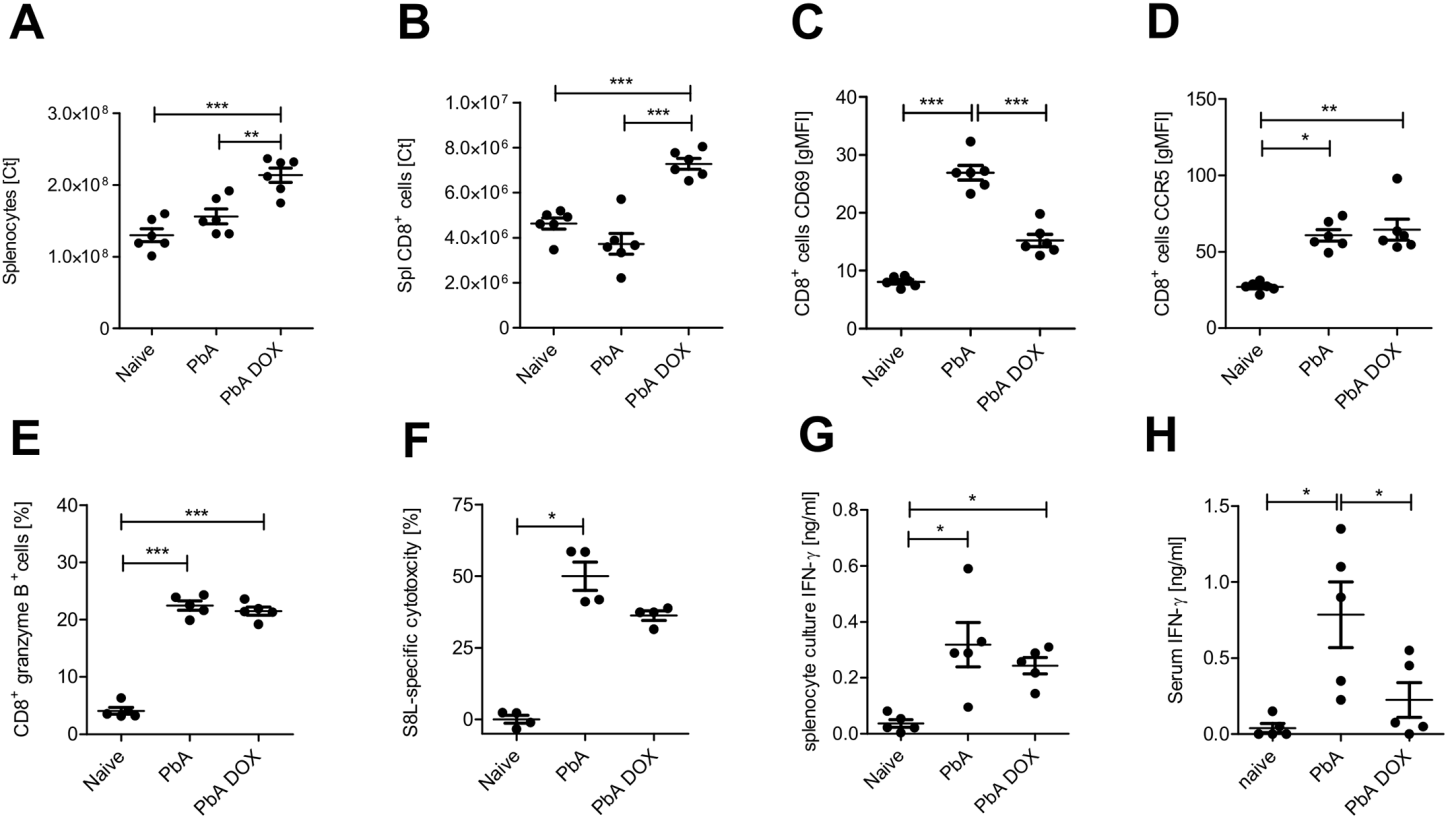
Splenic CD8^+^ T cell responses are altered after DOX administration in PbA infected mice. Analysis of splenocytes on dpi 6 in naïve, PbA ± 80 mg/kg/day DOX-treated C57BL/6 mice. **(A)** Total splenocyte number on dpi 6. **(B)** Absolute cell numbers of CD8^+^ splenocytes. **(C)** Geometric mean fluorescence intensity (gMFI) of CD69 on CD8^+^T cells. **(D)** gMFI of CCR5 on CD8^+^T cells. **(E)** Frequency of CD8^+^T cells expressing granzyme B intracellularly. **(F)** Parasite-specific lysis 18h after injection of PbA-infected ± DOX mice with SIINFEKL-pulsed syngeneic target cells. **(G)** *Ex vivo* IFN-γ production of splenocytes and **(H)** IFN-γ levels in serum from the same animals was measured by ELISA. One data set shows representative results from 2–3 independent experiments with 5–6 animals/group. Graphs display the mean and data were statistically tested for normal distribution and then with 1-way ANOVA and Tukey’s Post hoc test (A, B, C, E, G) or with Kruskal-Wallis followed by the Dunn’s post-hoc test, if data were not normally distributed (D, F, H). *p<0.05, **p<0.01,***p<0.001.

Priming and generation of immune effector cells in blood-borne diseases primarily takes place in the spleen [28]. Since the brains of DOX-treated PbA-infected mice contained less infiltrated T cells in comparison to untreated PbA-infected mice (Fig 2D and 2E), the DOX treatment might have an impact on effector cells, their local activity in the spleen and /or the cell recruitment into the brain. We therefore analysed the effector cell populations in the spleens of PbA-infected mice with and without DOX-treatment. Importantly, DOX-treated PbA-infected mice presented a splenomegaly with elevated total splenocyte counts as well as increased numbers of splenic CD8 T cells compared to spleens of naïve and PbA infected mice (Fig 3A and 3B and Figure A in S3 Fig).

Although surface expression of C-C chemokine receptor type 5 (CCR5) on splenic T cells (Fig 3D) and the frequency of T cells expressing intracellular granzyme B (Fig 3E) were not altered between DOX-treated mice and untreated PbA-infected mice, surface expression of CD69, a marker for general T cell activation, was significantly reduced on CD8^+^ T cells after DOX treatment (Fig 3C), suggesting a reduced T cell activation. Other markers that are associated with T cell activity were either found to be unchanged between PbA-infected groups with or without DOX treatment, such as PD-1 (Figure B in S3 Fig) or were slightly reduced after DOX treatment without reaching statistical significance, such as ICAM-1 and CD40L (Figures B and C in S3 Fig).

Next, we analysed the impact of the DOX-treatment on parasite-antigen-specific activities of splenic CD8^+^ T cells. Although the *in vivo* antigen-specific CTL lysis and the capacity of splenic CD8^+^ T cells from DOX-treated mice to produce IFN-γ was not significantly impaired (Fig 3F and 3H), in contrast, IFN-γ serum levels were decreased in DOX-treated mice compared to control-infected mice (Fig 3H), indicating a reduced level of Th1 cytokines.

Thus, although DOX-treated mice contained peripheral CD8+ T cells displaying a similar activation phenotype compared to T cells from untreated control-infected mice suffering from ECM, these T cells accumulated in their spleens and were hardly detectable in the brains—in contrast to untreated control mice. We conclude that this apparently impaired recruitment of effector cells upon DOX treatment—together with a reduced antigen load in the brain—contributed to an attenuated inflammation and ECM protection.

### Elevated parasite dose could not reverse DOX-mediated ECM protection

**Fig 4:**
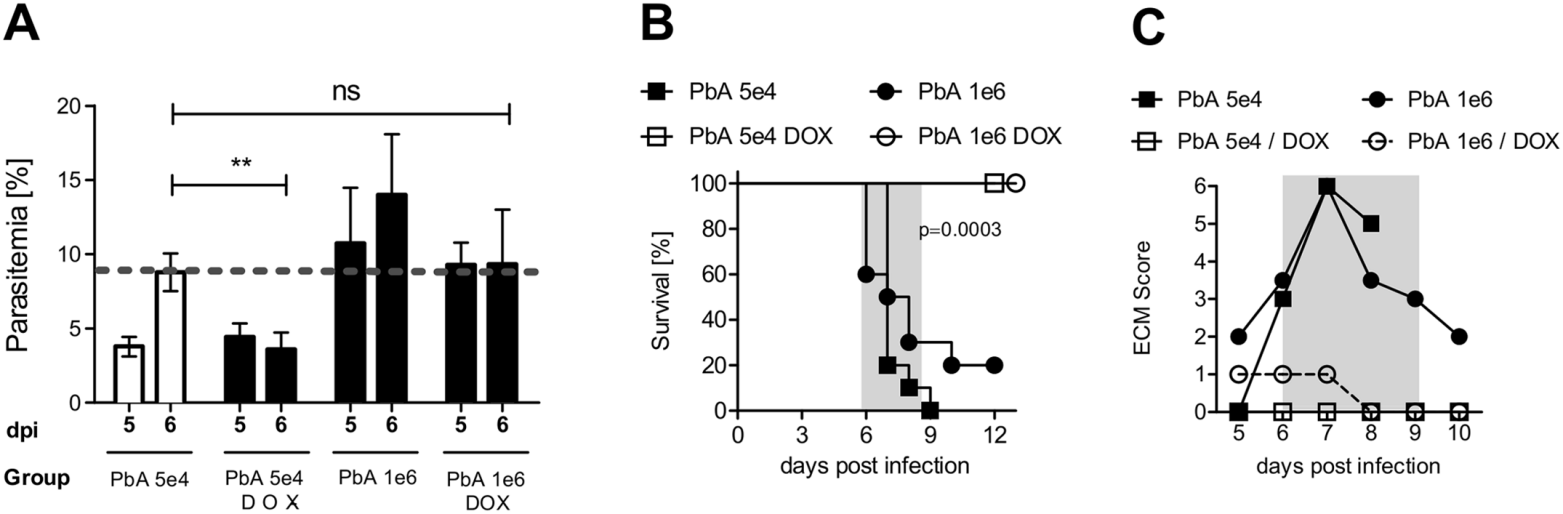
Elevated initial infective parasite dose could not reverse DOX-mediated ECM protection. C57BL/6 mice were infected with either 5*10^4^ or 1*10^6^ PbA-iRBCs and received from dpi 4–6 80 mg DOX/kg/day i.v. or not. (A) Blood parasite burden was assessed from dpi 5 on. (B) Survival and (C) ECM score (ECM phase dpi 6–9 marked in grey) were analyzed over the course of infection with low or high dose of parasites and DOX treatment. Representative data from 2–3 individual experiments with n = 10 mice/group are shown. Parasitemia is displayed as the mean/SD and ECM score as median. Survival data was analysed with log-rank (Mantel-Cox) test.

As we demonstrated above, DOX treatment of PbA-infected mice led to complete protection from ECM and also to an arrest of parasite growth on day 6 p.i. (Fig 4A), that is in line with its well-described anti-parasitic effects. We therefore addressed the question whether the impact of DOX-induced anti-inflammatory effects could be dissected from the anti-parasitic effects in our model. To rule out that the DOX-mediated decrease of parasitemia was solely responsible for the ECM protection, we determined the parasitemia of untreated PbA-infected mice on day 6, which is the time point of ECM onset. These control-infected mice displayed on dpi 6 an average parasitemia of 8% (Fig 4A).

Titration experiments regarding the infectious dose revealed that an infection with 1*10^6^ iRBC, which is a 20-fold increase of the parasite dose compared to the previous experiments, was sufficient to establish a parasitemia of approximately 8% on day 6 even after DOX treatment, thereby equivalent to the parasitemia of untreated mice infected with 5x10^4^ iRBC, which all died from CM (Fig 4A, see PbA 5e4 versus PbA 1e6 +DOX on 6 dpi). Survival and ECM development of mice that had received an elevated dose of parasites did not differ from those mice that had been infected with the lower dose of parasites that has been used in the other experiments (Fig 4B and 4C). Importantly, DOX administration promoted survival (Fig 4B) and inhibited ECM development in mice infected with either dose of PbA (Fig 4C). Furthermore, we demonstrated maintained BBB stability in DOX-treated mice infected with high dose PbA (S4 Fig). Thus, DOX treatment was able to protect PbA-infected mice from ECM even in the presence of an elevated parasite load, which strongly supports anti-inflammatory effects of DOX in addition to anti-parasitic effects.

Taken together, these results showed that DOX-induced protection from ECM was stable, indicating that DOX exerts strong immune-regulatory functions–in addition to its anti-parasitic effects—that inhibited disease progression by modulation of the inflammatory responses against PbA.

## Discussion

Here we show that the tetracycline DOX, in addition to its described anti-parasitic effects, is able to ameliorate inflammation in PbA-infected mice resulting in protection from ECM.

DOX targets a specific and essential organelle in *Plasmodium* parasites, the apicoplast, which is a bacterial remnant from a former endosymbiont, thereby inhibiting further development and division [13, 29]. A recent summary of optimized tetracyclines and their anti-malarial efficacy highlights the importance of tetracyclines as a prophylactic and treatment strategy in *Plasmodium* ssp. infection with a focus on its anti-parasitic effects that have been well known for a long time [30]. However, DOX has also been described to influence immune responses of the host [31] by dampening pro-inflammatory reactions in pathogen-induced inflammation [17, 32] and by reducing vascular degeneration [33]. This prompted us to investigate the capacity of DOX in protecting PbA-infected mice from ECM, a valuable translational model allowing detailed *ex vivo* analysis during an on-going infection. Indeed, we show here that daily intravenous administration of 80 mg/kg DOX starting on d+4 p.i. was able to protect PbA-infected C57BL/6 mice from BBB breakdown and prevented in an IL-10-independent manner the development of ECM (Fig 1 and Figure E is S1 Fig), which is mainly dependent on inflammatory cytokines such as granzyme B and cytotoxic T cells as supported by various animal studies [26].

Analysis of brain tissue by an RNA array revealed that DOX treatment of PbA-infected mice impaired the expression of chemokine, cytokine and adhesion molecules associated with inflammation and endothelial cell activation and BBB damage compared to control-infected mice. DOX treatment modulated CCL5, which is induced in the brain upon PbA infection [34]. This is in line with recent studies on post-mortem tissue samples from human patients that demonstrated an association of CCL5 with cerebral malaria. It was shown that samples from CM patients contained elevated levels of CCL5 and its cognate receptor CCR5 compared to non-CM controls [35]. Moreover, CCL5 apparently does not only act as a recruitment factor, it also has been related to the effector function of CD8^+^ T cells [36]. Deficiency in CCR5 or interferon gamma-induced protein 10 (IP10) both responsible for T cell recruitment under inflammatory conditions, confers resistance to ECM and reduces T cell migration to the brain [37, 38]. Importantly, Hirako et al. demonstrated recently the impact of CXCL9/10+ MO-DCs in the recruitment and activation of T cells, thereby promoting neuroinflammation and ECM [39]. The relevance of these chemokines in the DOX mediated protection will be interesting to address in the future. Especially CXCL10 is an interesting candidate as de Paula Costa et al. showed a reduction of its mRNA expression in cardiac tissue from *Trypanosoma cruzi* infected dogs that had been treated with doxycycline in combination with benznidazole [40].

Activated effector T cells have been accounted to play a decisive role in the disruption of the BBB and disease progression [41]. Parasite-specific cell-mediated responses increase systemic and local inflammation, leading to an activation of endothelial cells; genetically deficient mice or depletion strategies had impressively demonstrated the importance of adhesion molecules such the intercellular adhesion molecule 1 (ICAM-1), P-selectin on activated endothelial cells and leukocytes [42, 43] in the induction of ECM. In addition, local effector processes such as intravascular accumulation of leukocytes and CD8^+^ T cells [44] and the production of granzyme B [26] and perforin [45, 46] during ECM are well described. In particular, the recruitment of armed effector T cells to local tissue is discussed to be a critical step in ECM pathogenesis.

Antigen-specificity as well as effector function of T cells and a fully functional antigen-presentation have been shown to be indispensable for ECM induction [10, 26, 47]. A key finding was our results that PbA-infected mice that did not develop ECM after DOX treatment displayed slightly diminished activation in general (CD69, Fig 3C) but no significant reduction of cytotoxic activity of splenic CD8^+^ T cells (Fig 3D and 3G). Activation of T cells is essential for the induction of effector functions such as T cell cytotoxicity, which is a tightly regulated process and several signals like antigen-recognition, co-stimulation and cytokine-induced polarization are required [48]. Importantly, these effector T cells accumulated in the spleens of the DOX treated mice, whereas we observed reduced CD8^+^ T cell levels and less granzyme B production in the brains of DOX treated mice, pointing towards an influence of DOX on T cell migration and local activity compared to control-infected ECM-positive mice (Fig 2E). Importantly, a recent study by Shaw et al. demonstrated similar accumulation patterns of brain-infiltrating T cells in both ECM-inducing and ECM–non-inducing infections. They observed clustering of the T cells in perivascular spaces of the brains in both models; however, only in ECM-positive brains, a cognate interaction between T cells and cross-presenting endothelial cells occurred, probably leading to alteration of tight junction proteins and increased vascular permeability and death [49]. The authors suggested that in non-ECM infections, perivascular T cells fail to recognize their cognate antigen, restricting their pathogenic activity, including the secretion of cytotoxic mediators and thereby preventing ECM development. According to this hypothesis, we suggest that upon DOX treatment, communication between cells presenting parasite-derived antigen and CTLs in the brain might be impeded, also contributing to a mitigation of ECM.

In addition to granzyme B secretion by T cells, it could not yet be clarified whether additional differences may account for protection. Our data indicate an impact of DOX on T cell degranulation, since we observed significantly impaired granzyme B secretion in the brains of DOX treated mice in comparison to untreated animals. Several studies have shown that tetracyclines themselves can inhibit degranulation of stimulated antigen-specific T cells [50, 51], offering a possible mechanism by which cytotoxicity of activated T cells can be inhibited. Degranulation of fully activated antigen-specific T cells is highly dependent on vesicular trafficking [52]. Thus, it would be interesting to investigate in the future whether DOX indeed is able to inhibit degranulation in *Plasmodium* infection in more detail.

The stabilized BBB in ECM-negative DOX-treated mice was another central finding of our study. We observed an up-regulation and increased activity of gelatinolytic matrix metalloproteinases (MMPs) in brains of PbA-induced ECM-positive mice, whereas DOX-treated mice that were protected from ECM, contained reduced levels of MMP2 in their brains (Fig 2C). This supports the idea that enzyme activity may play a role in BBB disruption and disease progression i.e. by impaired access of T cells to the brain. MMPs are involved in endothelial cell and tissue damage, and both MMP2 and MMP9 have been proposed to be responsible for BBB disruption during *Plasmodium* infection [53] and described to be significantly inhibited by DOX [19]. MMP2 is constitutively expressed while the expression of MMP9 is inducible [54], which might explain differences in protein activity. Although we and others could show an up-regulation of either MMP-2 or MMP-9 during ECM pathology, MMP-9 deficient mice were similarly susceptible to ECM development as WT mice [55]. This, however, does not argue against a functional role of MMPs in ECM, since MMPs have overlapping functions and may compensate for each other.

DOX-mediated inhibition of matrix metalloproteinases (MMPs) and an amelioration of immune-mediated pathology were observed in several diseases and disease models like abdominal aneurysm, periodontitis, experimental autoimmune encephalomyelitis (EAE) and focal ischemia [18–21].

Importantly, MMP-2 is a molecule strongly linked to TNF, a well-known inducer of tissue degrading MMP-2 and MMP-9 in brain pericytes [56] and microglia [57]. Our experiments show that independently of cellular infiltrates and antigen presentation, TNF was strongly reduced on mRNA level and in primary brain tissue cultures after DOX treatment (Figure B in S2 Fig) and may therefore be responsible for changes in MMP-2 expression. Reduced levels of TNF superfamily members, indicating diminished local inflammation, could further contribute to the protection from ECM pathology [58, 59]. Thus, although a key role for the TNF superfamily in CM is controversially discussed, a contribution to the pathology by regulating MMP activities seems possible and supports its participation by secondary effects. Moreover, several studies describe minocycline, another tetracycline derivative, as having the ability to attenuate T cell-microglia interactions, cytokine production and MMP activity [20]. Minocycline also acts as an inhibitor of mononuclear phagocytes, and reduces TNF expression, proliferation and degranulation of CD8^+^ T cells [51]. This might contribute to the DOX-mediated protection, alongside other factors.

While DOX treatment led to a reduction in blood parasitemia, animals that had received a high parasitic dose to yield a similar parasitemia to that seen in ECM-positive untreated mice were still protected by DOX from ECM. These findings support the hypothesis that DOX exerts crucial effects on inflammatory immune reactions in this system, leading to protection of mice from ECM. A correlation with reduction of parasite mass indicated that DOX induced apparently a combination of anti-parasitic and anti-inflammatory effects. A recent study suggests that also artesunate may have additional anti-inflammatory properties in ECM treatment; since parasite numbers were reduced even stronger than after DOX, it is difficult to dissect both effects [60]. An immunosuppressive effect of artemisinin and its derivatives was also reported for several other diseases and experimental models, including arthritis, experimental colitis, systemic lupus erythematosus (SLE) and experimental autoimmune encephalitis (EAE) [61–64]. Importantly, this considerable finding should be analysed in more detail first in translational models to improve the understanding of the molecular action of these drugs as well as the therapeutic options for patients. Future approaches should also address the combined application of artesunate- the fast-acting first-choice antimalarial drug given i.v. in severe Malaria tropica—together with DOX in the ECM model to examine a potential beneficial impact on disease course after ECM onset, closer to severe malaria and relevant for treatment of diseased humans, beyond our intervention studies at an early stage of disease.

In conclusion, the data emphasize the importance of translational models and suggest that DOX treatment of *Plasmodium* infection should be beneficial for the infected host through anti-inflammatory, BBB-stabilizing and MMP-inhibiting properties in addition to anti-parasitic effects that could work in concert with fast-acting anti-malarial drugs given in combination. Retrospective meta-analyses might elucidate the immunomodulatory impact of DOX and other anti-malarials that have only been correlated with an anti-parasitic effect so far.

A better understanding of molecular mechanisms occurring during cerebral malaria and therapeutic interventions by detailed drug research may result in better treatment strategies for patients infected with *P*. *falciparum* and other CM patients in the future.

## Material and methods

### Ethical statement

The study includes work with experimental animals and was designed under consideration of the 3R rules and the ARRIVE checklist [65]. Mouse studies were approved by the local regulatory agency Landesamt fuer Natur, Umwelt und Verbraucherschutz (LANUV (84–02.04.2012A295). Research staff was trained according to FELASA B guidelines. Female 6–8 weeks old C57BL/6 N mice (average weight of 20g) were purchased from Janvier (Le Genest Saint Isle, France). IL-10 ko mice [66] were bred in the House of Experimental Therapy (HET) of the University of Bonn according to institutional animal guidelines. All mice were housed in autoclaved Tecniplast^®^ cages under specific pathogen free conditions with common animal bedding. A 12 hour light/dark cycle was maintained and water and food were provided ad libitum. Mice were allocated randomly to cages with n = 4–6 mice per group accordingly to the individual experimental groups. Survival experiments were performed with 10 mice per group and *ex vivo* experiments were performed with n = 4–6 animals per group and 2–3 times repeated, accordingly to sample size determination performed before by statistical power calculation. Infection, treatment and assessment of the health status were performed sequentially.

Longterm anaesthesia for *ex vivo* analysed experimental mice was given before perfusion by intramuscular injection of 10μl Rompun^®^ (2%, Bayer, Germany) + 40μl Ketamin (50mg/ml, Ratiopharm GmbH Germany) per 25g mouse. In order to meet humane endpoints, critically sick mice were euthanized by cervical dislocation under isofluran inhalation anaesthesia.

### Parasites, infection and disease assessment

In all experiments, *P*. *berghei* infected red blood cells (iRBCs) were used to inoculate mice. Mice had an average weight of 20 g and were infected intravenously (i.v.) with 5x10^4^ iRBCs or 1x10^6^ iRBCs (high dose), obtained from mice that had been infected intra-peritoneally (i.p.) with stock solution. Stock solution contained 1x10^7^ iRBCs in glycerine and was stored in liquid nitrogen. Stock mice were of the same background as experimental animals and parasites were isolated on d5 p.i. Experiments were conducted with either *Plasmodium berghei* ANKA (PbA) [67] or *P*, *berghei* expressing OVA (PbTG) [10], the latter being kindly provided by Rachel Lundie. All experiments were conducted with *P*. *berghei* ANKA except analysis of *in vivo* cytotoxicity, IFN-γ release and detection of antigen-specific T cells (PbTG). Experimental setup and used *Plasmodium* strains are denoted in the figure legends. From day 4 post infection (p.i.), mice were punctured at the tail vein for daily blood-smears. Before day 4, parasite levels were almost undetectable (d1 and d2 p.i.) or very low (d3 p.i.). For evaluation of disease onset, mice were monitored twice daily for ECM symptoms. ECM development was scored according to the following symptoms: 0 = without symptoms, 1 = ruffled fur, 2 = hunching, 3 = wobbly gait, 4 = limb paralysis, 5 = convulsions, 6 = coma adapted from Amante et al. [68]. Mice did not succumb to infection or any other cause with a score of ≤4; mice that reached a score of >4 were sacrificed immediately accordingly to predefined ethical criteria. Graphs display a timeframe in which ECM development occurs, all infected animals were sacrificed by dpi 20 or immediately upon development of hyperparasitemia or anaemia.

### Treatment and drug administration

If not otherwise stated mice were treated i.v. with 80 mg/kg DOX dissolved in 200 μl PBS daily starting from d4 till d6 p.i. Mice had an average weight of 20 g. Oral (p.o.) treatment was performed with a special gauge, mice received 80 mg/kg or 250 mg/kg DOX dissolved in 200 μl H_2_O.

### Perfusion and lymphocyte isolation

In order to remove non-adhering and non-infiltrating erythrocytes and blood-lymphocytes long-term anaesthetised mice were intracardially perfused for 5 minutes with 1x PBS. Spleens and brains were removed, placed in MACS buffer (1x PBS containing 1% FCS and 2 mM EDTA) or collagenase A buffer (Sigma-Aldrich, St. Louis, USA), respectively, and placed on ice. Spleens were gently pressed through a sieve, washed with MACS buffer and centrifuged (10 min., 1500 rpm), cell numbers were determined and adjusted to 2x10^7^ per ml medium, and 50 μl were plated into 96 well plates. OVA-peptide S8L was added in a final concentration of 2 μM to spleen cell culture. Brain tissue was cut into small pieces and incubated in 2 ml collagenase A buffer for 30 min. at 37°C in a water bath. After homogenization in MACS buffer single cell suspensions were then centrifuged at 1500 rpm for 8 min. at 4°C. 500 μl of primary brain cells were adjusted to 2.5x10^7^ cells per ml RPMI and 100/μl plated into 96 well plates for cell culture. The cell pellet was suspended in 5 ml 30% percoll that was then layered onto a two-step percoll gradient made of 3 ml 37% percoll and 3 ml 70% percoll. Samples were centrifuged at room temperature for 20 minutes without brakes at 2000 rpm. After percoll separation, two white interphases were isolated and transferred into a fresh tube and washed with MACS buffer. After a second centrifugation step, cells were resuspended in 300 μl FACS buffer (1x PBS, 1% FCS) and counted.

### *In vivo* cytotoxicity, FACS analysis and ELISA

Cytotoxic T cell activity was determined as described before [69]. Briefly, five days post infection splenocytes from naive syngeneic donor mice were prepared, split into equal populations, loaded with or without 1μM OVA peptide S8L (SIINFEKL) and with CFSE (1 μM or 0.1 μM respectively). Both populations were mixed at equal numbers, every mouse received 1x10^7^ of total cells. 18h later, mice were sacrificed and spleens were isolated. CFSE positive cells were acquired by flow cytometry and both cell populations were determined. The specific lysis was determined as follows: 100-(ratio sample/ratio average of naive) x 100. Splenocytes and brain leukocytes were stained and analyzed for expression of CD4, CD8, CD11b, CD11c, CD45, CD69, CCR5 and granzyme B in a FACS Canto II flow cytometer (BD, San Jose, USA).

Data were analysed with FlowJo Software (Treestar Inc., Ashland, USA). ELISA IFNγ (e-Bioscience, San Diego, USA), TNF, CCL5 and Granzyme B (R&D, Minneapolis, USA) ELISA-kits were used according to the manufacturers protocol. The TNF antibody has not been tested by the company for crossreactivity with lymphotoxin (LT).

### RNA-isolation, RNA-array and RT-PCR

Brain tissue samples were collected from all groups six days post infection, where some of the control mice already showed signs of cerebral pathology. Samples were snap frozen in liquid nitrogen and stored at -80 degrees until RNA extraction. Samples were transferred to tubes with 1.4mm Precellys-Ceramic beads and homogenized in TRI-Reagent^®^ (Sigma-Aldrich, St. Louis, USA) with the Precellys^®^ 24 (PEQLAB, Erlangen, Germany) homogenizator. After bromchlorpropane/isopropanol isolation samples were treated with DNase and RNA quantity and quality was examined by the OD260 value.

One microgram of total RNA was reverse transcribed to cDNA.

IFN-γ forward: TCAAGTGGCATAGATGTGGAAGAA,

IFN-γ reverse: TGGCTCTGCAGGATTTTCATG.

PbA 18srRNA forward: CTAACATGGCTTTGACGGGTA,

PbA18srRNA reverse: TGTCACTACCCTCTTATTT.

PCRs were performed with the Rotor- Gene 3000 or 6000 (Corbett Research, Hilden, Germany) with conditions set to: 15 min at 95 °C followed by 45 cycles of 15 s at 94°C, 20 s at 58 °C and 20 s at 72 °C. Rate of temperature change was 0.8 °C/s. Copy numbers were determined by a reference plasmid. CCL3 and CCL4 RNA levels were measured with the Qiagen Quantitect Primer-Assay and the Qiagen RotorGene SybrGreenkit (Qiagen, Hilden, Germany). For SABioscience (SABioscience, Hilden, Germany) RT- RNA-Array cDNA samples were isolated with the above described protocol but prior to DNase digestion purified with the Qiagen RNeasy Mini Kit. Samples were eluted in 40 μl RNase free Water and the quality of the samples were spectrometrically tested and ribosomal RNA band integrity measured by the Experion (Bio-Rad, Hercules, USA).1 μg RNA was transcribed into cDNA with the RT-First Strand Kit from SABioscience according to the manufacturer’s protocol. Samples were stored at -20°C until usage. We performed the Mouse Endothelial Cell Biology RT-Profiler PCR Array from SABioscience in a 96-Well-Plate as described in the corresponding protocol. The sample was mixed with RT-SYBR Green / ROX qPCR (SABioscience) and added to the plate; the PCR was performed on a LightCycler 480 from Roche (Basel, Switzerland). Analysis of the array was done with the PCR Array Data Analysis Web Portal from SABioscience (http://www.SABiosciences.com/pcrarraydataanalysis.php). Analysis was performed in R version 3.1.0. Pba and Dox samples were compared using Mann-Whitney statistic, a non-parametric test without assumptions on the distribution of Ct values. Adjustment for multiple testing was performed by Benjamini-Hochberg correction.

### Zymography

Half of a mouse brain was homogenized with 1.4 mm Precellys-Ceramic beads with the Precellys^®^ 24 (PEQLAB, Erlangen, Germany homogenized material was centrifuged in a fresh tube for 5 minutes at 12 000 g at 4°C. 0.5 ml of the supernatant was incubated with 50μl of gelatine-Sepharose 4B (GE-Healthcare, Munich, Germany) for 1 hour at 4°C. After a brief centrifugation at 500 g for 2 minutes supernatant was washed 3 times with working buffer (50 mmol/L Tris-HCl, pH 7.6; 150 mmol/L NaCl; 5 mmol/L CaCl2; 0.05% BRIJ-35; 0.02% NaN3; 1% Triton X-100). Incubation of the supernatant with 150 μl elution buffer (working buffer + 10% DMSO) for 30 minutes at 4°C, followed by 2 minutes centrifugation at 500 g. 15 μl sample together with 2x SDS loading buffer and a prestained standard (Novex^®^ Sharp Pre-stained Protein Standard; Invitrogen, Paisley UK) (8μl + 5μl loading buffer) were applied to a SDS-page gel containing 0.5% gelatine (Sigma-Aldrich, St. Louis, USA). The gel was run at 125 V for 1.5 h. 1 h incubation in renaturing buffer (2.5% TritonX 100), followed by 30 minutes incubation at RT in developing buffer (10 mmol/L Tris-Base; 50 mmol/L Tris-HCl; 150 mmol/L NaCl; 5 mmol/L CaCl2; 0.05% BRIJ-35; adjusted to pH 7.6). Replace buffer with fresh developing buffer and incubate for 48h at 37°C. Stain overnight with 0.5% coomassie stain. Destain for 1 hour with 30% Methanol. For quantification of relative gelatinolytic activity the optical density of bands was analyzed with FIJI graphic software. Scanned images were converted into gray scale pictures and the colours were reversed before a black and white picture was generated. The size of the bands was analyzed and normalized to the naive group (fold change against naive).

### Cross-presentation assay of brain microvessels

Brain microvessels were isolated as described before [27]. Briefly, the brain was minced, homogenized and fractionated by 15% dextran gradient centrifugation. Microvessels in the pellet were retained on a 40 μm cells strainer. After collagenase and DNaseI digestion for 90 min, the microvessels were plated on to a filter plate. LR-BSL8.4 cells are transduced with a T cell receptor (TCR) recognizing the SQLLNAKYL epitope from *P*. *berghei* and contain a NFAT-lacZ reporter cassette. After overnight coculture of LR-BSL8.4 cells and microvessels the wells were stained with X-gal and after 6 hours blue spots were imaged and counted in an ImmunoSpot Analyser.

### Statistical analysis

Survival was analysed with the Mantel-Cox log-rank test. ECM scores are displayed as median. Parasitemia is displayed as mean/SD.

PCR-array analysis was performed in R version 3.1.0. PbA and PbA/DOX samples were compared using Mann-Whitney statistic, a non-parametric test without assumptions on the distribution of Ct values. Adjustment for multiple testing was performed by Benjamini-Hochberg correction.

If the data met criteria for normal distribution, they were tested with one-way ANOVA followed by the Tukey post-hoc test to check differences between groups. Non-parametric data were tested with Mann-Whitney U test or with the Kruskal-Wallis test followed by the Dunns post-hoc test to correct for multiple groups. For data organization and statistical analysis, Graphpad^®^ Prism 5.0 software was used.

## Supporting information

S1 Fig Parasitemia is similar in PbA-infected mice before treatment start. (A) C57BL/6 mice received 5*10^4^ PbA-infected erythrocytes (PbA-iRBC). Before start of the DOX treatment on 4 dpi, blood smears were taken from the tail vein to determine blood parasitemia by Giemsa staining. After confirmation that all mice were similarly infected, we started the DOX treatment. (B) Parasitemia was analyzed on 6 dpi in both PbA infected groups (±DOX) as described in (A). (C) Quantitative RT-PCR of *Plasmodium berghei* ANKA 18S rRNA in brain tissue of naïve, untreated or DOX treated PbA infected animals on 6 dpi. Data in A, B, C are displayed as mean and statistically analyzed with Mann-Whitney U test; p<0.05 was considered significant. (D) Course of parasitemia in DOX treated PbA-infected animals (E) WT C57BL/6 mice and IL-10^-/-^ mice received 5*10^4^ PbA-infected erythrocytes (PbA-iRBC). Indicated groups received 80mg DOX/kg from dpi 4–6. N = 8–10 per group. Survival of all infected mice was monitored and analysed with log-rank (Mantel-Cox) test.
(PDF)

S2 Fig No reduction of CCL5, MMP-9 enzyme activity, IFN-γ, or cross-presenting activity after DOX treatment. Determination of CCL5 (A) and TNF (B) secretion from brain homogenates isolated from brains of naïve and PbA-infected mice ±DOX via ELISA. (B) On dpi 6, supernatants of overnight cultures from isolated primary brain cells were analyzed for TNF family. Representative graphs of two independent experiments with 4–5 mice/group are shown. (C) Six days post PbA infection brain tissue of C57BL/6 naïve mice and infected ± 80 mg/kg/day DOX were examined for MMP expression/ activity and granzyme B production. For enzymatic analysis brain tissue was subjected to zymography. Gelatine zymography of brain tissue extracted on day 6 p.i. (D) Quantification by scanning densitometry of the gelatinolytic bands of proMMP-9. Representative experiments are shown with 6–7 mice/ group. Relative scanning units of MMP-9 are shown as fold change against expression of naïve animals displayed as median and statistically tested with the Kruskal-Wallis-test followed by the Dunns post-hoc test. On day 6 p.i. cells from brains of naïve or PbA-infected mice with or without DOX treatment were identified as CD8^+^ T cells with the help of flow cytometry and further analyzed for granzyme B frequency (E) and gMFI (F). (G) Cross-presentation assay from brain microvessels. Naïve, untreated or DOX-treated mice were sacrificed on day 6 post infection. Brain microvessels were isolated and co-cultured with LR-BSL8.4 reporter cells to detect cross-presentation of a PbA epitope. Blue spots stained with X-gal were counted. Data were log-transformed to meet parametric standards. Graphs represent one of 2–3 independent experiments with 5–6 animals/group. Data are displayed as mean and statistically analyzed with 1-way ANOVA followed by Tukey’s post-hoc test. *p<0.05 after analysis of normal distribution.
(PDF)

S3 Fig Activation markers in splenic T cells. (A) Frequency of splenic CD8^+^T cells of naïve and PbA-infected mice ±DOX on day 6 p.i. These splenic CD8^+^T cells were further analyzed by flow cytometry for PD-1 (B), ICAM-1 (C) and CD40L (D).
(PDF)

S4 Fig Evans blue assay in PbA (high) infected mice after DOX treatment. Brains of PbA (low) and PbA (high) ± DOX mice were analyzed on day 6 p.i. for BBB integrity with the help of an Evans Blue assay. All groups of mice received i.v. 2% Evans Blue dye in NaCl, which was allowed to circulate in the blood for one hour. Thereafter, brains were harvested and incubated in formamide for 48h. Extravasation of the dye into the brain was quantified by measuring the absorbance of the dye that had been extracted by formamide at 620nm. Data are displayed as mean and statistically analyzed with Mann-Whitney U test. p<0.05 was considered significant.
(PDF)

S1 Table Mouse endothelial cell biology RT-Profiler PCR Array. RNA samples from brain tissue of naive (n = 2), PbA ± DOX (n = 4) were subjected to the array. Brain tissue of groups were extracted on 6 dpi, RNA was prepared and analysed via PCR array. Data is expressed as fold change. Results from statistical analysis (p-values and adjusted p-values) are provided for both the comparison between the PbA-infected groups ± DOX.
(PDF)

## Abbreviations

ACT: artemisinin-based combination therapies
APCs: Antigen-presenting cells
BBB: blood-brain barrier
CCL5: chemokine (C-C motif) ligand 5
CCR5: C-C chemokine receptor type 5
CM: cerebral malaria
CNS: central nervous system
DCs: dendritic cells
ECM: experimental cerebral malaria
i.v.: intravenously
ICAM-1: intercellular adhesion molecule 1
IFN-γ: interferon gamma
IL: interleukin
IP10: interferon gamma-induced protein 10
iRBCs: infected red blood cells
MMP: matrix metalloproteinase
p.i.: post infection
PbA: *Plasmodium berghei* ANKA
PbTg: *Plasmodium berghei* ANKA expressing transgenes
TNF: tumour necrosis factor
