# Supplementary figures and images for: Doxycycline inhibits experimental cerebral malaria by reducing inflammatory immune reactions and tissue-degrading mediators
Source: PLoS One. 2018 Feb 13;13(2):e0192717. doi: 10.1371/journal.pone.0192717 (PMC5811026; doi:10.1371/journal.pone.0192717)

S1 Fig

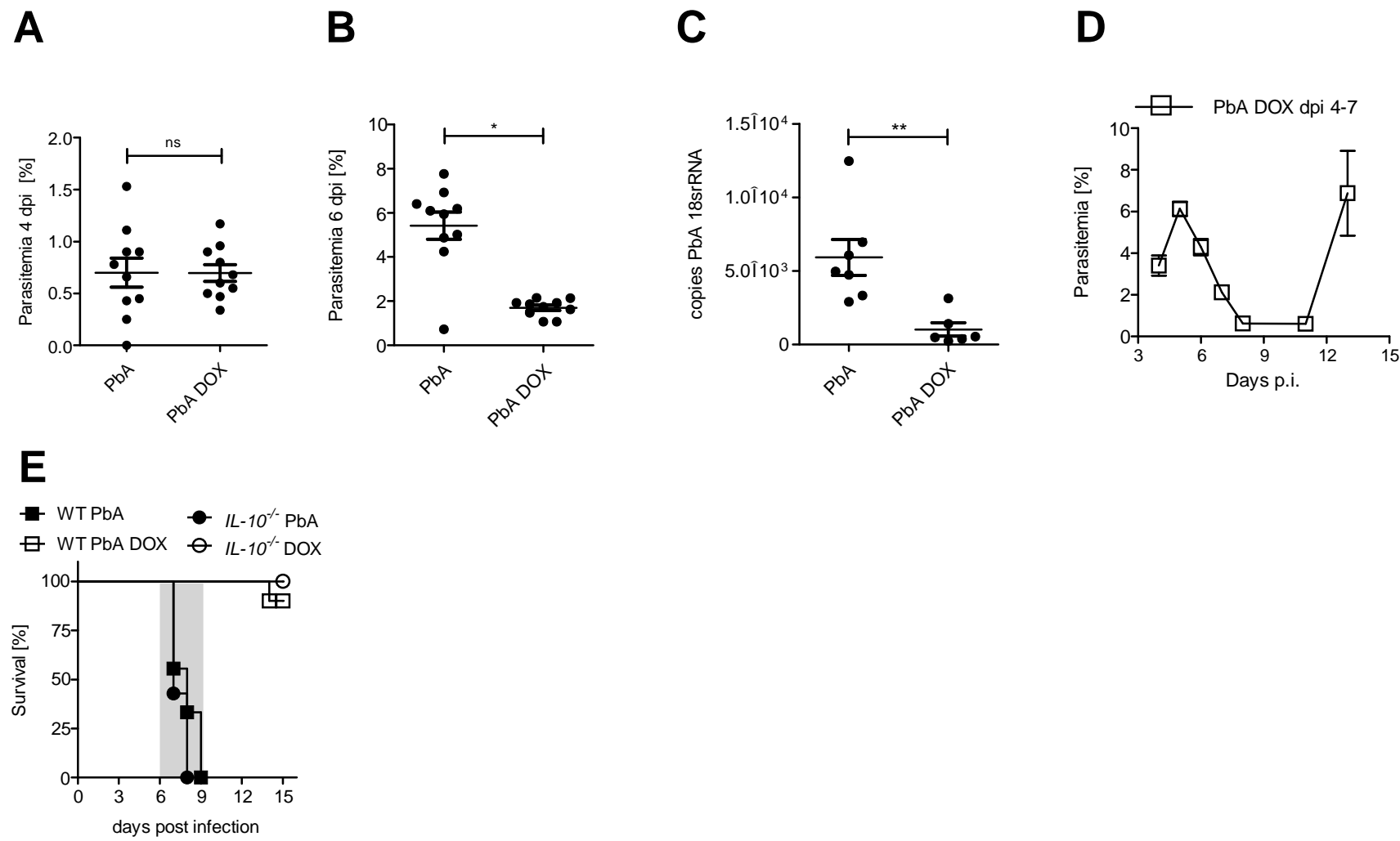

Supplement: S1 Fig — (A) C57BL/6 mice received 5*104 PbA-infected erythrocytes (PbA-iRBC). Before start of the DOX treatment on 4 dpi, blood smears were taken from the tail vein to determine blood parasitemia by Giemsa staining. After confirmation that all mice were similarly infected, we started the DOX treatment. (B) Parasitemia was analyzed on 6 dpi in both PbA infected groups (±DOX) as described in (A). (C) Quantitative RT-PCR of Plasmodium berghei ANKA 18S rRNA in brain tissue of naïve, untreated or DOX treated PbA infected animals on 6 dpi. Data in A, B, C are displayed as mean and statistically analyzed with Mann-Whitney U test; p<0.05 was considered significant. (D) Course of parasitemia in DOX treated PbA-infected animals (E) WT C57BL/6 mice and IL-10-/- mice received 5*104 PbA-infected erythrocytes (PbA-iRBC). Indicated groups received 80mg DOX/kg from dpi 4–6. N = 8–10 per group. Survival of all infected mice was monitored and analysed with log-rank (Mantel-Cox) test. (PDF) [file pone.0192717.s001.pdf]

**A**

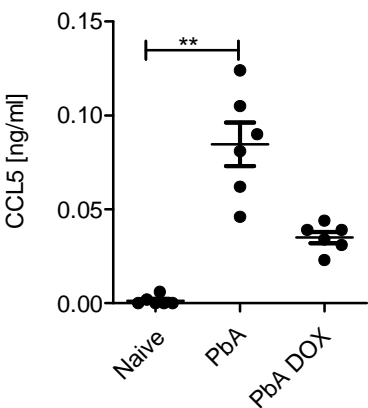

# B

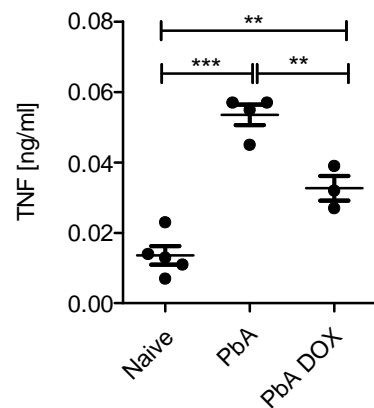

**C**

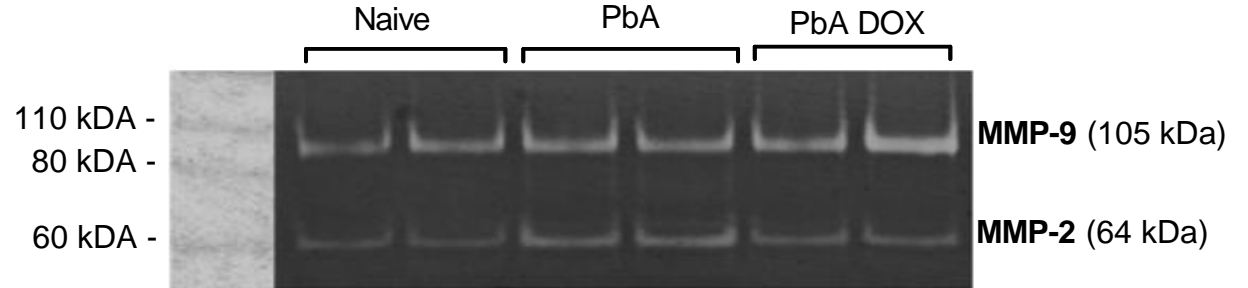

# D

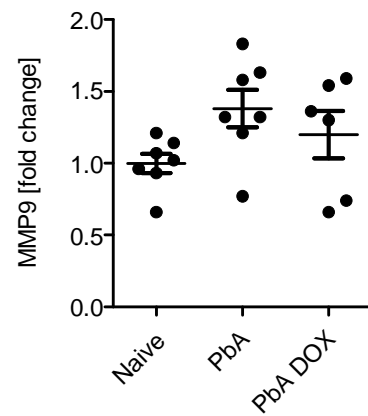

# E

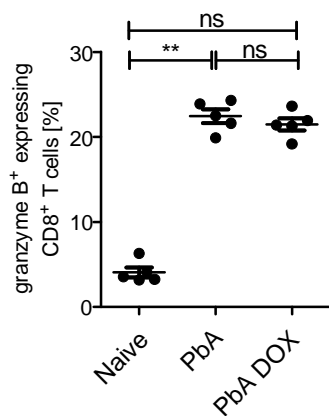

# F

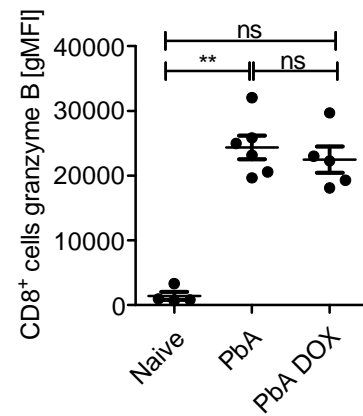

## G

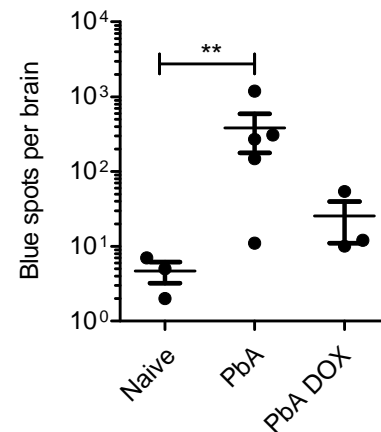

Supplement: S2 Fig — Determination of CCL5 (A) and TNF (B) secretion from brain homogenates isolated from brains of naïve and PbA-infected mice ±DOX via ELISA. (B) On dpi 6, supernatants of overnight cultures from isolated primary brain cells were analyzed for TNF family. Representative graphs of two independent experiments with 4–5 mice/group are shown. (C) Six days post PbA infection brain tissue of C57BL/6 naïve mice and infected ± 80 mg/kg/day DOX were examined for MMP expression/ activity and granzyme B production. For enzymatic analysis brain tissue was subjected to zymography. Gelatine zymography of brain tissue extracted on day 6 p.i. (D) Quantification by scanning densitometry of the gelatinolytic bands of proMMP-9. Representative experiments are shown with 6–7 mice/ group. Relative scanning units of MMP-9 are shown as fold change against expression of naïve animals displayed as median and statistically tested with the Kruskal-Wallis-test followed by the Dunns post-hoc test. On day 6 p.i. cells from brains of naïve or PbA-infected mice with or without DOX treatment were identified as CD8+ T cells with the help of flow cytometry and further analyzed for granzyme B frequency (E) and gMFI (F). (G) Cross-presentation assay from brain microvessels. Naïve, untreated or DOX-treated mice were sacrificed on day 6 post infection. Brain microvessels were isolated and co-cultured with LR-BSL8.4 reporter cells to detect cross-presentation of a PbA epitope. Blue spots stained with X-gal were counted. Data were log-transformed to meet parametric standards. Graphs represent one of 2–3 independent experiments with 5–6 animals/group. Data are displayed as mean and statistically analyzed with 1-way ANOVA followed by Tukey’s post-hoc test. *p<0.05 after analysis of normal distribution. (PDF) [file pone.0192717.s002.pdf]

S3 Fig

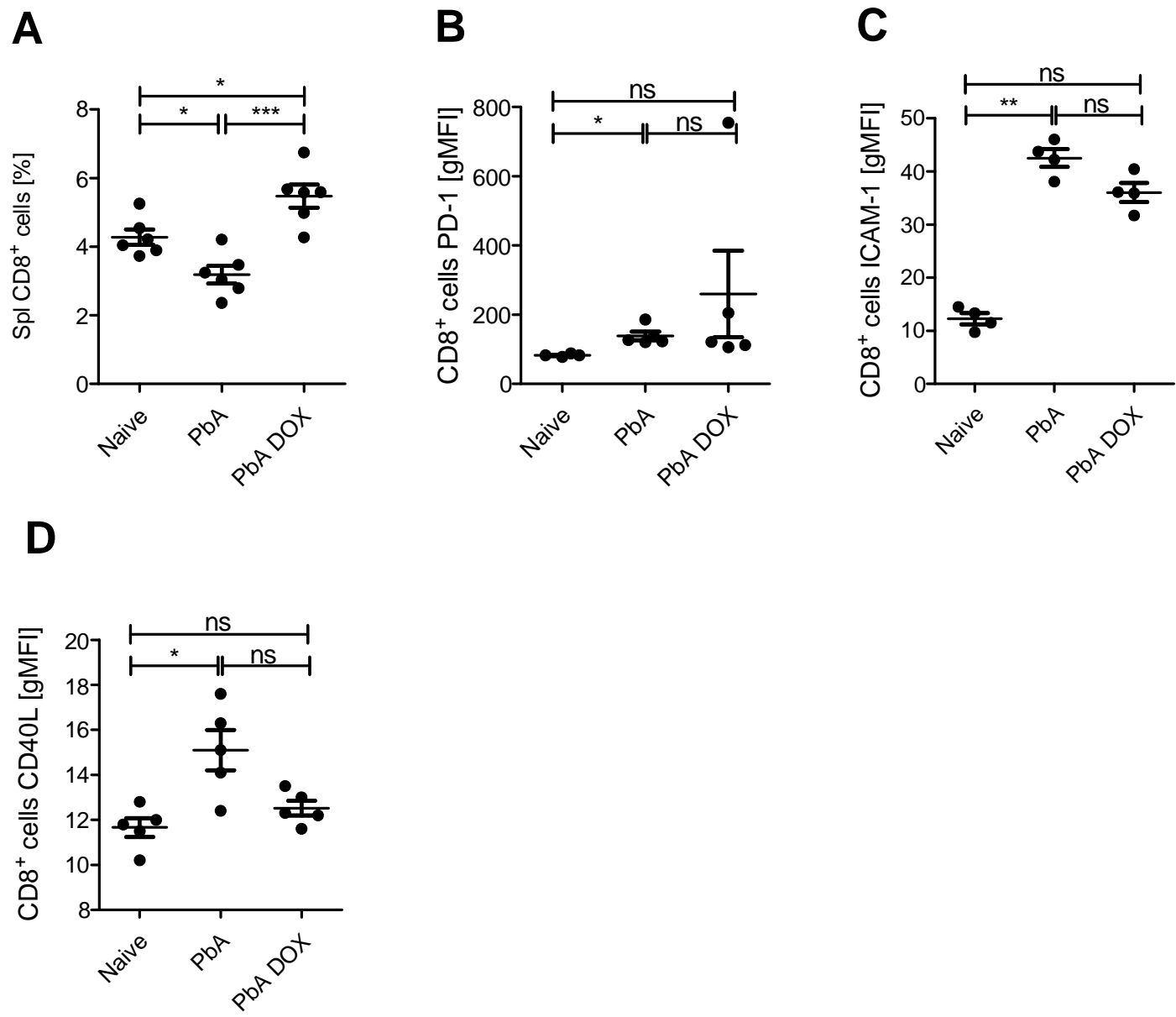

Supplement: S3 Fig — (A) Frequency of splenic CD8+T cells of naïve and PbA-infected mice ±DOX on day 6 p.i. These splenic CD8+T cells were further analyzed by flow cytometry for PD-1 (B), ICAM-1 (C) and CD40L (D). (PDF) [file pone.0192717.s003.pdf]

S4 Fig

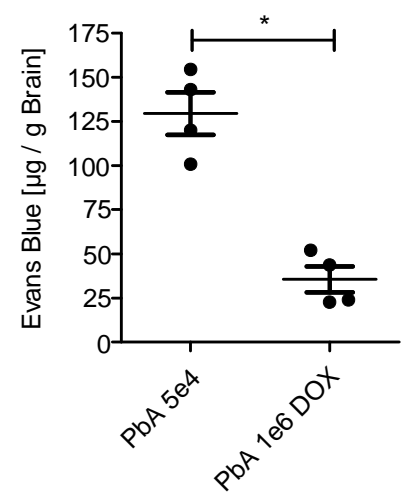

Supplement: S4 Fig — Brains of PbA (low) and PbA (high) ± DOX mice were analyzed on day 6 p.i. for BBB integrity with the help of an Evans Blue assay. All groups of mice received i.v. 2% Evans Blue dye in NaCl, which was allowed to circulate in the blood for one hour. Thereafter, brains were harvested and incubated in formamide for 48h. Extravasation of the dye into the brain was quantified by measuring the absorbance of the dye that had been extracted by formamide at 620nm. Data are displayed as mean and statistically analyzed with Mann-Whitney U test. p<0.05 was considered significant. (PDF) [file pone.0192717.s004.pdf]
