# Supplementary material for: Doxycycline inhibits experimental cerebral malaria by reducing inflammatory immune reactions and tissue-degrading mediators
Source: PLoS One. 2018 Feb 13;13(2):e0192717. doi: 10.1371/journal.pone.0192717 (PMC5811026; doi:10.1371/journal.pone.0192717)
Supplement: S1 Table — RNA samples from brain tissue of naive (n = 2), PbA ± DOX (n = 4) were subjected to the array. Brain tissue of groups were extracted on 6 dpi, RNA was prepared and analysed via PCR array. Data is expressed as fold change. Results from statistical analysis (p-values and adjusted p-values) are provided for both the comparison between the PbA-infected groups ± DOX. (PDF) [file pone.0192717.s005.pdf]

S1 Table

Gene	Fold change Naive vs. PbA	Fold change Naive vs. PbA DOX d4-d6	Fold change (p-value, adjusted p-value) PbA vs. PbA DOX d4-d6
Ccl2	76,40	10,77	-7,09 (0,021, 0,160)
Sell	28,31	9,71	-2,92 (0,021, 0,160)
Cxcl2	24,60	4,64	-5,30 (0,021, 0,160)
Ccl5	16,69	6,27	-2,66 (0,021, 0,160)
Sele	14,03	11,23	-1,25 (0,564, 0,728)
Vwf	12,84	81,27	6,33 (0,386, 0,570)
Tnf	12,63	3,84	-3,29 (0,248, 0,417)
Selp	10,04	3,15	-3,19 (0,043, 0,214)
Icam1	8,71	4,78	-1,82 (0,021, 0,160)
Il6	7,33	1,63	-4,50 (0,021, 0,160)
Tnfsf10	5,48	6,59	1,20 (0,564, 0,728)
Cxcl1	5,18	2,07	-2,50 (0,248, 0,417)
Mmp9	4,23	4,71	1,11 (0,564, 0,728)
Serpine1	3,59	1,39	-2,59 (0,083, 0,269)
Il1b	2,82	1,79	-1,57 (0,083, 0,269)
Casp1	2,57	1,60	-1,60 (0,021, 0,160)
Cdh5	2,30	1,31	-1,76 (0,021, 0,160)
Fasl	2,25	1,46	-1,54 (0,149, 0,404)
Fas	2,17	2,29	1,05 (0,773, 0,832)
Birc2	2,16	2,28	1,06 (1,000, 1,000)
Itga5	2,05	1,29	-1,59 (0,021, 0,160)
Csf2	1,96	1,07	-1,83 (0,083, 0,269)
Tgfb1	1,96	1,33	-1,47 (0,021, 0,160)
Nos3	1,95	1,50	-1,30 (0,248, 0,417)
Pecam1	1,93	1,80	-1,07 (0,149, 0,404)
Thbs1	1,93	1,03	-1,86 (0,083, 0,269)
Edn2	1,75	1,04	-1,68 (0,248, 0,417)
Plg	1,71	1,21	-1,42 (0,149, 0,404)
Tnfaip3	1,70	1,12	-1,51 (0,043, 0,214)
Cxcr5	1,63	1,08	-1,51 (0,083, 0,269)
Vcam1	1,62	2,08	1,28 (0,149, 0,404)
Vegfa	1,50	1,83	1,22 (0,248, 0,417)
Ifnb1	1,47	1,08	-1,36 (0,083, 0,269)
Selplg	1,42	1,00	-1,41 (0,043, 0,214)
Nos2	1,42	1,09	-1,30 (0,564, 0,728)
Plat	1,37	1,15	-1,19 (1,000, 1,000)
Fn1	1,32	1,55	1,18 (0,564, 0,728)
Edn1	1,31	1,39	1,06 (0,773, 0,832)
Cflar	1,31	1,30	-1,01 (0,564, 0,728)
Ptgis	1,30	1,19	-1,09 (0,386, 0,570)
Ripk1	1,30	1,13	-1,15 (0,083, 0,269)
Pgf	1,27	-1,08	-1,37 (0,248, 0,417)

Gene	Fold change Naive vs. PbA	Fold change Naive vs. PbA DOX d4-d6	Fold change (p-value, adjusted p-value) PbA vs. PbA DOX d4-d6
Nppb	1,25	-1,23	-1,53 (0,083, 0,269)
Thbd	1,22	1,02	-1,20 (0,248, 0,417)
Agt	1,19	1,51	1,27 (0,248, 0,417)
Rhob	1,15	1,07	-1,07 (0,386, 0,570)
Angpt1	1,14	1,51	1,33 (0,083, 0,269)
Ace	1,13	1,17	1,04 (0,773, 0,832)
Itgb1	1,10	-1,02	-1,13 (0,021, 0,160)
Il11	1,10	-1,14	-1,26 (1,000, 1,000)
Bcl2l1	1,08	1,08	1,00 (0,248, 0,417)
Tfpi	1,07	1,18	1,11 (0,248, 0,417)
Flt1	1,03	1,63	1,58 (0,043, 0,214)
Itgav	1,01	-1,01	-1,02 (0,564, 0,728)
Pdgfra	1,00	1,00	-1,00 (0,665, 0,832)
Bcl2	-1,01	1,13	1,14 (0,248, 0,417)
Adam17	-1,03	-1,01	1,02 (0,773, 0,832)
Col18a1	-1,05	1,00	1,05 (1,000, 1,000)
Il3	-1,05	-1,18	-1,13 (0,248, 0,417)
Casp3	-1,05	1,23	1,30 (0,248, 0,417)
Mmp1a	-1,05	-1,08	-1,03 (0,773, 0,832)
Ednra	-1,06	1,13	1,19 (0,386, 0,570)
Naip1	-1,06	-1,17	-1,10 (0,773, 0,832)
Bax	-1,10	-1,17	-1,07 (0,248, 0,417)
Sod1	-1,10	-2,00	-1,82 (1,000, 1,000)
Npr1	-1,11	-1,17	-1,05 (0,773, 0,832)
Fgf1	-1,12	1,01	1,13 (0,248, 0,417)
Cx3cl1	-1,12	-1,13	-1,01 (0,773, 0,832)
Anxa5	-1,13	-1,17	-1,03 (0,773, 0,832)
Plau	-1,15	-1,02	1,13 (0,386, 0,570)
Il7	-1,16	1,06	1,23 (0,248, 0,417)
Casp6	-1,16	-1,03	1,13 (0,248, 0,417)
Kit	-1,18	-1,21	-1,03 (0,773, 0,832)
Cradd	-1,20	-1,08	1,11 (0,248, 0,417)
Ocln	-1,24	-1,08	1,16 (0,386, 0,570)
Itgb3	-1,25	-1,30	-1,04 (1,000, 1,000)
Timp1	-1,30	-1,09	1,18 (0,248, 0,417)
Cpb2	-1,31	-1,12	1,17 (0,386, 0,570)
Mmp2	-1,37	-1,39	-1,02 (0,773, 0,832)
Agtr1a	-1,50	-1,02	1,46 (0,149, 0,404)
Tymp	-1,54	-1,69	-1,10 (0,564, 0,728)
Kdr	-1,56	1,09	1,71 (0,043, 0,214)
Tek	-1,81	1,06	1,92 (0,043, 0,214)
Pf4	-1,89	-1,89	1,00 (0,773, 0,832)
